# The Pulmonary Surfactant: Impact of Tobacco Smoke and Related Compounds on Surfactant and Lung Development

**DOI:** 10.1186/1617-9625-2-1

**Published:** 2004-03-15

**Authors:** J Elliott Scott

**Affiliations:** 1 Lung Development Section, Biology of Breathing Group, Manitoba Institute of Child Health & Departments of Oral Biology and Anatomy, Faculties of Dentistry and Medicine, University of Manitoba, Winnipeg, Manitoba, Canada

## Abstract

Cigarette smoking, one of the most pervasive habits in society, presents many well established health risks. While lung cancer is probably the most common and well documented disease associated with tobacco exposure, it is becoming clear from recent research that many other diseases are causally related to smoking. Whether from direct smoking or inhaling environmental tobacco smoke (ETS), termed secondhand smoke, the cells of the respiratory tissues and the lining pulmonary surfactant are the first body tissues to be directly exposed to the many thousands of toxic chemicals in tobacco. Considering the vast surface area of the lung and the extreme attenuation of the blood-air barrier, it is not surprising that this organ is the primary route for exposure, not just to smoke but to most environmental contaminants. Recent research has shown that the pulmonary surfactant, a complex mixture of phospholipids and proteins, is the first site of defense against particulates or gas components of smoke. However, it is not clear what effect smoke has on the surfactant. Most studies have demonstrated that smoking reduces bronchoalveolar lavage phospholipid levels. Some components of smoke also appear to have a direct detergent-like effect on the surfactant while others appear to alter cycling or secretion. Ultimately these effects are reflected in changes in the dynamics of the surfactant system and, clinically in changes in lung mechanics. Similarly, exposure of the developing fetal lung through maternal smoking results in postnatal alterations in lung mechanics and higher incidents of wheezing and coughing. Direct exposure of developing lung to nicotine induces changes suggestive of fetal stress. Furthermore, identification of nicotinic receptors in fetal lung airways and corresponding increases in airway connective tissue support a possible involvement of nicotine in postnatal asthma development. Finally, at the level of the alveoli of the lung, colocalization of nicotinic receptors and surfactant-specific protein in alveolar cells is suggestive of a role in surfactant metabolism. Further research is needed to determine the mechanistic effects of smoke and its components on surfactant function and, importantly, the effects of smoke components on the developing pulmonary system.

## Introduction

Tobacco in various forms, as well as tobacco-related compounds such as marijuana, represent agents that present serious and insidious health risks to the general population. Both of these drugs have long and interesting histories. As this review is focused primarily on tobacco, marijuana use will be discussed only as it reflects on health effects resulting from both tobacco and marijuana. Tobacco use passed into Europe in the late sixteenth century after initial encounters between Europeans and native North and South Americans [1]. Tobacco was seen often as a medicine. Several well known European physicians extolled the virtues of tobacco as a medicinal herb [1] and tobacco enemas were recommended for treatment of cholera and to loosen the bowels [2]. Ironically, one of among some twenty ailments purportedly amenable to tobacco was cancer [2]. For the next two centuries modest changes in cultivation, largely in the American colonies, provided increasing supplies of tobacco to Europe although it should be noted that consumption was taken largely in the form of chewing plugs [2], snuffed, or smoked in pipes [3]. It was not until the late 1860's that a sudden change in consumption occurred. In fact the change was startling. According to Tilley [4], in 1869 about 2 million cigarettes were being manufactured in the United States and it was uncommon to see someone smoking in public. Some ten years later with the advent of new curing methodologies, the introduction of the Bonsack cigarette-making machine, and as the cigarette fashion took hold, 300 million units were produced. Indeed, the Bonsak machine could produce some 100,000 cigarettes a day, the equivalent of the work of 30–40 labourers. These machines marked an innovative turning point for the tobacco industry [5]. The production level initiated by the automated machines was reflected in the consumption trend as tobacco sales between the late nineteenth century until the end of the first World War underwent a major shift as 50% of sales were accounted now by cigarettes rather than pipe tobacco [6]. With this remarkable shift to cigarettes and the concurrent increase in smoke inhalation compared to snuffed or chewed tobacco, deaths due to lung cancer showed dramatic increases [7].

The long history of both tobacco and marijuana as addictive drugs, their common routes of exposure, and their many common components make for an interesting dilemma in the health care field for both common and different reasons. On the one hand, the detrimental effects of cigarette smoking through both primary and secondary routes of exposure have become clear over the past few years [8]. The list of potential health risks is large and continues to grow after prolonged years of tobacco exposure. On the other hand, there is a general movement toward the legalization and use of marijuana, particularly for medicinal purposes. Ironically marijuana smoking presents many of the same risks as tobacco smoking; this is largely ignored in the public press. Surveys of public opinion suggest that marijuana use is generally considered relatively innocuous. Yet many of the same components that make tobacco such a health risk are present in marijuana smoke. These components are associated with elevated risk of heart disease, ovarian cancer, bone cancer, breast cancer, pancreatic cancer, oral cancers, bladder cancer, and of course lung cancers. Indeed the separation of marijuana-induced health risks from tobacco-induced risks is difficult, as most users are dependent on both drugs as well as potentially other more potent drugs. Since the respiratory tree and lungs are the first areas of exposure to these agents, the interactions of smoke components with the cells, lining fluids and materials of the lungs are of considerable interest. A great deal has been written about potential smoke effects on many of the component cells of the conducting and respiratory tissues within the lung. While it is clear that smoking is by far the greatest risk factor associated with development of lung cancer [9,10], the diversity of products in tobacco and marijuana smoke, the large number of pulmonary cell types and the complex environment of the lung make the delineation of smoke-induced diseases very difficult.

## The Pulmonary System

The pulmonary system which encompasses not only the lungs but the conducting airways, the nasal cavities, nasopharynx, oropharynx and larynx, is probably the most complex system in the body. This is due to the fact that the pulmonary system provides the most intimate interface with the external environment of any region of the body. The surface area of the lung tissue, approximately 120 m^2 ^by recent estimates [11-13], represents the largest body surface area exposed to the environment. At the level of the alveoli where gas exchange occurs, the biological barrier presents as an extremely attenuated interface composed of the cell membranes and fused basal laminae. At the same time this arrangement must provide protection against a vast range of biological and non-biological elements. This is obviously a difficult undertaking. This complex environment must in part account for the large numbers and variations of cell types detected in the conducting airways and respiratory tissues [14]. Some 40 different cell types have been described in the lining tissues, bronchial tree and respiratory tissues [14]. Their functions have only begun to be elucidated and their relationships to the complex disease processes that affect the lungs have only begun to become clear. Within this context, recent research has shown that pulmonary surfactant is a major player both in terms of the intrinsic function of the lungs as well as presenting a first line of defense against immunological, biological and non-biological threats [15-17]. Indeed as Phelps points out, every organism or particle that enters the pulmonary system in the inspired air comes into contact with the pulmonary surfactant [18]. Thus in addition to its surface tension lowering capabilities, surfactant undoubtedly plays a number of important roles, such as mounting of an immunological defense or activating intrinsic cellular responses.

Before turning to a discussion of the interaction of tobacco smoke and related agents with the pulmonary surfactant system, it is important to have a conceptual knowledge of exactly what composes the lung surfactant. Therefore, we will begin our discussion in the form of a short review of what our latest concepts are concerning the surfactant, its composition and function. It should be noted, however, that this short review is not intended to be exhaustive as extensive reviews are available by acknowledged experts on pulmonary surfactant (see references for a complete issue devoted to the pulmonary surfactant [19,20]).

## The Pulmonary Surfactant

Within the lung, an aqueous lining layer exists to varying degrees within the alveoli and intrapulmonary duct system [21]. The composition and characteristics of this layer are critical to many lung functions, for example gas exchange, defense against microorganisms and pulmonary compliance. Estimates of the thickness and volume of this layer suggest 25 mls of total liquid for an average 70 kgs of body weight resulting in a thickness of probably less than 0.1 μm [22]. Within this layer the pulmonary surfactant exists and interfaces between the alveolar air and lining liquid phases. The pulmonary surfactant is an extremely complex mixture of components which fall generally into two broad categories. The complexity of these components reflects the corresponding complex functional role of the surfactant and indeed the multifunctional aspects of the mix are unique in the body as they reflect the extracellular role as well as the intracellular regulatory aspects of the surfactant. The major components of lung surfactant are phospholipids. It has been generally held for many years that the surface-active properties of surfactant lie in the domain of these components. However, it has become clear over the last decade that such a distinction is not as clear as it was once held to be, since the pulmonary surfactant proteins and their inherent characteristics are not simply left over by-products of some other system such as the blood vascular system. Rather the proteins are both specific and instrumental in pulmonary surfactant function. These two characteristic components, the phospholipids and proteins, will be dealt with individually and their impact on function discussed below briefly.

## Phospholipid Components and their Contribution to Function

Excellent recent reviews of the biosynthesis, composition and functional contributions of the lipid components of the pulmonary surfactant are available [23,24] and the reader is referred to these for detailed descriptions of the surfactant. The present review will provide only a cursory overview as a foundation to discussing the effects of smoke inhalation on the surfactant.

**Figure 1:**
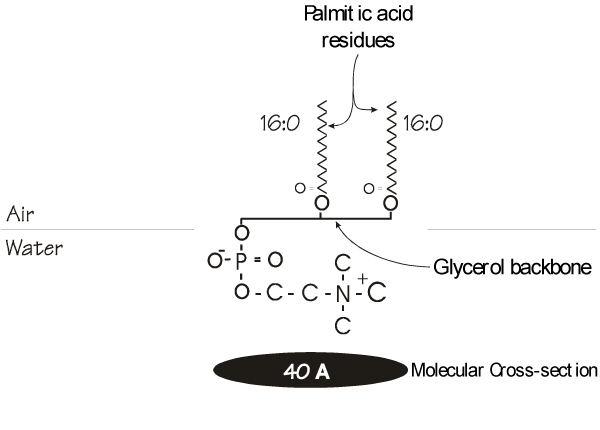
**Arrangement of a molecule of dipalmitoylphosphatidylcholine (DPPC) at the interface of the air and an aqueous hypophase**. The fatty acid moieties are displaced away from the polar water phase while the polar head group associates with the water. The uniformity of the fatty acid chains allows tight packing of adjacent DPPC molecules providing a small cross-sectional profile.

The pulmonary surfactant is composed of approximately 90% lipids [25,26] with the remainder being proteins specifically, and somewhat uniquely, associated with functional aspects of the lipids. These will be discussed below. The lipid fraction is a complex mix of which the majority is phospholipid, accounting for about 85% of the total. The remaining fraction, composed of neutral lipids, contains some trace amounts of triglycerides and fatty acids [27] but its main component is cholesterol which may have some important functions [28,29]. Of the phospholipid fraction, phosphatidylcholine accounts for 70–80% of the total [30]. While these components are not particularly unique in themselves, several features of the surfactant are indeed peculiar and of course reflect its specialized function within the lung. Of particular note is the fatty acid moieties esterified to the glycerol backbone within the phosphatidylcholine fraction. Although it may be difficult to characterize a certain fatty acid complement as being typical given the complexity and huge number of possible combinations, biochemical references espouse a general 1-saturated, 2-unsaturated configuration for phospholipids arranged on the glycerol backbone at least for those components contributing to cell membrane bilayers [31]. However, the pulmonary surfactant displays a unique subfraction within the phosphatidylcholine fraction. In particular, disaturated phosphatidylcholine (DSPC) almost all of which is dipalmitoyl (Figure 1) accounts for between 60% and 70% of the total [20,32], although it should be noted that some estimates suggest DSPC levels lie more in the 40% range [33]. Such discrepancies may be related to maturity, species or experimental techniques. This latter possibility is discussed in detail by Goerke (1998). In fact, recent studies support the contention that species differences do exist and these may relate to the functional or evolutionary background of the surfactant in question. For example, the levels of DSPC as a percentage of total phospholipid in surfactant show a relative increase through vertebrate evolution while the ratio of cholesterol to total phospholipid seems to decline [34]. Functional changes may also be reflected in surfactant composition. In dunnarts, an Australian heterothermic marsupial, induction of a state of torpor is associated with increases of both the ratios mentioned above [35,36]. Similarly lungs of certain air-breathing fish have phospholipids that are severalfold less saturated than those of reptiles and mammals [37]. Thus, as these authors point out, the presence of high surfactant cholesterol levels, which may occur rapidly in as little as two hours [38], may suggest a protosurfactant which evolved as a means of controlling surface viscosity in ectothermic animals [28].

The defining characteristic of lung surfactant is its ability to generate very low surface tension at minimum expansion. Surface tension may be thought of as the force that resists expansion of a liquid [36]. This is due to the attraction of the molecules for each other. Experiments with normal air-filled lungs or lungs rinsed with detergent show that the alveolar area decreases with increasing surface tension, suggesting an equilibrium exists in the lung between surface and tissues forces [39]. In terms of the surface forces, from the LaPlace equation which relates the pressure (P) across a sphere such as the lung alveolus to the sphere's radius, r (P = γ × 2/r, γ is the surface tension coefficient), as the radius decreases, if the surface tension was fixed, the transpulmonary pressure, P would have to increase [40,41]. This unstable situation is avoided by lining the alveoli with a surface film which, as alveolar volume decreases allows reduction of the surface tension, γ, enabling the alveoli to reach stability. It is generally accepted that DSPC, and specifically that fraction which is composed of dipalmitoylphosphatidylcholine (DPPC), is primarily responsible for the surface tension lowering abilities of the pulmonary surfactant [42] and in fact is probably the only phospholipid capable of generating the very low surface tensions observed upon film compression [23]. This phospholipid displays a gel to liquid phase transition temperature of 41°C, and thus exists in an ordered gel state at body temperature (37°C) which has implications for its function as well as raising questions as to composition and spreadability in the alveolus [43]. This arrangement together with the uniformity associated with the saturated palmitic acid moieties esterified to the glycerol backbone allows DPPC to pack closely at the air-water interface within the alveolus and, after reorganization into multi-layered surface pools [44,45] to withstand the high compressive pressures during exhalation allows the surfactant to achieve very low surface tensions in the alveolus.

The other surfactant phospholipid of particular note is phosphatidylglycerol (PG). This phospholipid is present at undetectable to very low levels in cells and is predominantly involved in synthesis of cardiolipin (di-phosphatidylglycerol) associated with the inner mitochondrial membrane [46]. During fetal lung development, PG is maintained at low levels but, depending on the species, either immediately before or at birth PG levels in the surfactant rise until about 10% of the surfactant is accounted for by this phospholipid. Conversely the levels of phosphatidylinositol (PI) decline reflected the common intermediate in synthesis of PG and PI, CDP-diacylglycerol [47]. While the stimulus for this shift in synthesis of fetal lung from PI to PG near term is not clear, the elevated presence of PG in amniotic fluid has for some years been the basis of a diagnostic test to evaluate fetal lung maturity [48,49]. It should be noted that recent assessments using such parameters as lamellar body counts (the intracellular storage form of surfactant) [50] or ultrasound [51] may ultimately replace PG levels as diagnostic tools. The function of PG within the pulmonary surfactant is not clear. Administration of myoinositol, which induces a depletion of surfactant PG and a corresponding elevation of PI, does not seem to alter the efficiency of the pulmonary surfactant [52,53]. Nevertheless, recent evidence suggests that the acidic phospholipids such as PG within the surfactant may interact with certain of the surfactant proteins (SP-B in particular) and may be related to selective adsorption of DSPC and reorganization of these phospholipids from the monomolecular film at the air-water interface of the alveolus [42,43,54].

Other minor phospholipids and neutral lipids exist in the pulmonary surfactant and while it is too early to dismiss their contributions as negligible, relatively few investigations into their functions have appeared. The reader is referred to recent reviews for more details [23,55].

## Proteins

Four major proteins exist in the pulmonary surfactant. While they were recognized some years ago, it is only within the last few years that their functions have begun to be elucidated. Initially the proteins were thought to be derived in part from serum and there was considerable disagreement as to their number and function [56]. Today it is clear that several of these proteins are unique and all perform some basic function related to the processing, integration, reutilization, and probably other related functions of the surfactant. Surfactant proteins, termed SP-A, SP-B, SP-C and SP-D, have been localized to the surfactant obtained by bronchoalveolar lavage and all have been shown to perform some critical function ascribed to surfactant. Briefly, SP-A and SP-D are hydrophilic Ca^+2^-dependent proteins, both of which appear to originate from the surfactant-producing type II alveolar cell (see next section) and possibly the non-ciliated (Clara) cells of the bronchial epithelium [57]. SP-A is the most abundant of the surfactant proteins. It is highly conserved and has been identified in the lung of many species [58]. It displays collagenous and globular regions and is a member of a family of proteins termed collectins [59]. In lung lavage SP-A exists as a large oligomeric glycoprotein of about 650 kDa in size [60] while the protein monomer has a molecular mass of 28–36 kDa [61] depending on the degree of post-translational modification [61,62]. The function of SP-A is still under debate and in fact it probably performs multiple functions, one being to act, along with SP-D, as broad spectrum alveolar opsonins [63]. However, its important nature in the surfactant is suggested by studies that demonstrated SP-A interacts with lipid mono- and bilayers [64], restores biophysical properties to oxidized surfactant [65] and mediates uptake of phosphatidylcholine by type II alveolar cells [66]. Furthermore, potential interactions of SP-A with SP-B in formation of tubular myelin, the symmetrical phospholipid arrays intervening between the secreted lamellar bodies and the air-liquid monolayer has been demonstrated [67,68], suggesting vital importance of these proteins to the surfactant. Ironically, recent studies with SP-A knockout mice, while showing some changes in surfactant properties, do not support a critical requirement in lung for SP-A as postnatal survival and pulmonary function were not altered [67,69].

SP-D, a collagenous 43 kDa glycoprotein secreted by type II alveolar cells [70], has also been identified in tracheal submucosal glands [57], the gastric antrum [71] and several other glands. SP-D consists of four homotrimeric units whose primary translation products are greatly enriched in the surfactant-producing type II alveolar cells [72]. A number of potential functions of SP-D have been suggested [73]. Particularly noteworthy in this regard is the ability of SP-D to interact and participate in the clearance of microorganisms including influenza A by binding to oligosaccharides of hemagglutinin [74,75]. Within the surfactant, the contribution of this protein to function is not entirely clear. There has been some suggestion SP-D may have a particular affinity for phosphatidylinositol [76] if presented in the correct fashion, however, Taneva and colleagues (1997) could not demonstrate a head-group preference in surface-balance studies and attributed SP-D interactions with phospholipid to hydrophobicity [77]. Nevertheless, mice with an SP-D null (SP-D -/-) genotype develop emphysema in the presence of elevated lung DSPC levels [78], indicating that this surfactant protein does have some critical functions at the alveolar level. Finally a very important observation in SP-D (-/-) mice that induced expression of rat SP-D corrects pulmonary surfactant abnormalities through a cell signaling mechanism [79] suggests SP-D regulates surfactant metabolism in type II alveolar cells [80].

The remaining two surfactant proteins, SP-B and SP-C, are generally considered to be hydrophobic in nature and thus associate readily with the phospholipids of the surfactant. SP-B is expressed by both type II alveolar cells and the nonciliated bronchiolar epithelial Clara cells [81,82]. Its function in the latter cells is not clear. In the lavage SP-B exists as a homodimer of about 18 kDa [83]. Unlike the water soluble proteins, SP-B associates with phospholipid bilayers through amphipathic regions generated by the three dimensional α-helical association of polar and non-polar residues [84]. Evidence suggests that SP-B reacts to increasing surface tension by reorientation of its subunits into phase with each other [85] enabling reversible rapid lipid insertion into the air-liquid monolayer [43]. SP-B may also be involved in surfactant secretion through regulation of directionality related to apical and basal membranes of the type II cells as well as in formation of tubular myelin [86]. Hereditary deficiency of SP-B in human infants and in mice is associated with severe lethal respiratory distress [87,88].

SP-C exists as a very non-polar α-helical protein of approximately 4.2 kDa composed of a mixture of isoforms (see review by Johansson [89]). It appears to be the only surfactant protein exclusively expressed by lung tissue [82] and is the only surfactant protein detected exclusively in the surfactant producing type II cells [90]. SP-C is altered posttranslationally by the addition of two palmitoyl groups to cysteines-5 and -6 [89]. Changes in the degree of palmitoylation may be associated with some pathological conditions such as alveolar proteinosis [91]. In the surfactant, the amphipathic nature of the α-helix may allow the protein to orient in a transmembrane way in bilayers of dipalmi-toylphosphatidylcholine and dipalmitoylphosphatidylglycerol [92]. However, in the surfactant the α-helical portion of the protein may occur such that the axis is almost parallel with the air-liquid interface [45,93]. The palmitoylation phenomenon may influence the rapidity with which phospholipid within the subphase reservoir is recruited to the monolayer [94,95]. However, the complex nature of the surfactant-subphase relationship at differing surface pressures may require or induce a variety of secondary structure conformations including extended β-sheets [85] despite evidence that SP-C is quite stable [96].

## Source of the Surfactant and Functional Considerations

**Figure 2:**
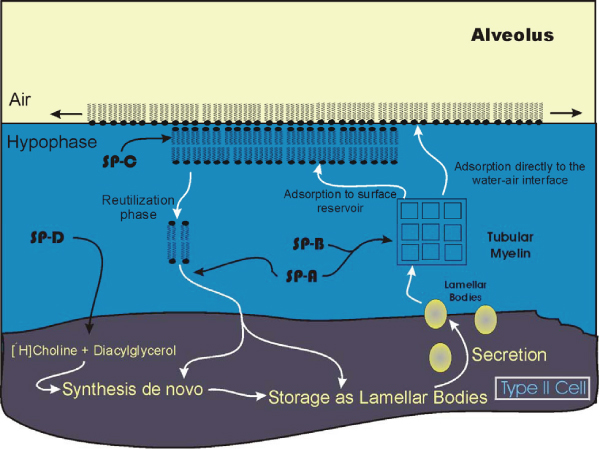
**Scheme showing the route for de novo surfactant (DPPC) synthesis, secretion as lamellar bodies, transposition to tubular myelin and reutilization of components in lung type II cells**. Phospholipids are represented by filled circles (polar head group) with fatty acid chains projecting into the air away from the polar hypophase. Potential examples of single sites of surfactant protein function (SP-A, SP-B, SP-C and SP-D) are shown; multiple functions and sites are likely. New concepts concerning the surface reservoir are indicated by the multiple phospholipid layers at the air-liquid interface [45,105]. Note the extensive extracellular processing of the surfactant as it is secreted at the apical surface of the type II cell, the air-hypophase interface and reutilization.

Some 40 or more pulmonary cell types have been identified [14], making the lung by far the most heterogeneous of organs. However, at the level of the terminal respiratory units, the alveoli, a relatively simple cellular composition is present. The major lining cells which present an extremely large surface area to inhaled air and therefore any toxins are the type I alveolar cells [97]. This cell type covers some 90% of the gas exchange area and is very susceptible to injury [98]. Interspersed among the type I cells are cuboidal type II alveolar cells. These cells are very active metabolically and, together with the non-ciliated bronchiolar (Clara) cells, are the source of the majority of the components of the surfactant [99-101]. Details of the biosynthetic processes whereby surfactant phospholipids are formed and the hormonal control of this process is beyond the scope of the present discussion; the reader is referred to numerous reviews [19,24,100,102,103]. The major components of the surfactant, once formed within the type II cells, are incorporated into lamellar bodies which therefore represent the intracellular storage form of the surfactant [104]. These osmiophilic bodies are easily identifiable in type II cells and the complex regulatory mechanisms whereby they are secreted are in part, worked out [55,106]. In addition to synthesis and secretion, over the last decade it has become apparent that major quantities of the pulmonary surfactant, after being released into the hypophase and sorted to the monomolecular phospholipid layer which lies at the air-liquid interface, are reutilized [107-109] (Figure 2). In neonates, as much as 90% of the surfactant is reutilized [110]. The processing of the extracellular material within the hypophase in the alveolus can be divided into several different stages reflecting the biophysical characteristics of the surfactant. Separation of the various forms by centrifugation [111,112] has shown a spectrum of attributes measurable by density, surface activity and infrared signatures [113,114]. As these fractions exist extracellularly, exposure to smoke or other environmental pollutants may directly alter the processing at various stages of this extracellular material [115,116]. Furthermore changes in processing and reuptake may be associated with some diseases such as pulmonary alveolar proteinosis [59].

DPPC, as the primary phospholipid of the surfactant, is generally considered to display the surface tension characteristics required to withstand the high surface pressures that occur during the exhalation phase of the respiratory cycle [33]. However due to the high gel transition temperature of DPPC (41°C), a film of this material would exist in a solid gel state at body temperature. Thus, for the film to be spreadable at 37°C, addition of cholesterol or other unsaturated phospholipids is necessary [28]. Within the alveolus, the lining surfactant has traditionally been considered to exist as a thin continuous layer. The application of modern imaging techniques such as cryo-scanning or transmission electron microscopy have supported this view and shown a thin continuous layer in the alveolus [117]. In this model, interaction of phospholipids and proteins within the alveolus accounted for the low surface tension and through a process of "squeeze-out" the monomolecular film at the surface air-liquid layer was believed to be enriched in DPPC [118] allowing the generation of surface tension values observed for the lung surfactant. However, observations using the most recently developed instrumentation such as scanning force or atomic force microscopy which allow visualization at the molecular level provide strong evidence that this theory is inaccurate [45,105,119,120]. Rather, recent views suggest the existence of multiple layered regions in a non-homogeneous surface surfactant [21]. The multi-layered regions unfold successively during inhalation and collapse into the layered form again upon expiration. These multilayers have been visualized [105]. The hydrophobic surfactant proteins B and C in particular may be intimately involved in organizing this process [45,120] and appear to be segregated in a liquid expanded compared to a condensed phase [121,122]. Thus surfactant metabolism involves not only the steps of intracellular synthesis and secretion, but multiphase complex transitions within the hypophase between various compartments. This allows many potential sites for interaction or interference with the surface tension-generating characteristics of the surfactant which may ultimate be reflected in stability of the alveolus.

## Tobacco Smoke and Lung Surfactant

From the foregoing brief description of the pulmonary surfactant system, it is clear that the surfactant and its microenvironment present an extremely complex face, both in terms of composition and function, to any materials reaching the respiratory tissues. At the level of the air exchange tissue, the pulmonary surfactant presents the first interface encountered by inhaled smoke, whether from tobacco, marijuana or any other inhaled aerosolized material, for example fungal spores, mites or other allergens. Tobacco use and the potential for exposure to environmental tobacco smoke (ETS) has become an extremely important issue over the last several decades as it has become apparent that smoking carries with it a major and also preventable risk for the development of lung cancer. Lung cancer accounts for about 30% of all cancer deaths in men and 20% of all cancer deaths in women [10]. In the United States, lung cancer accounts for 20% of all economic costs related to cancer treatments. This massive burden on the health care system is largely due to the continuing exposure to cancer-causing agents in tobacco smoke. Indeed some 80% of lung cancer deaths can be attributed to smoking [9,123,124]. The issue of susceptibility to induction of smoking-related lung cancer is a complex problem and beyond the scope of the present review. The reader is referred to recent compilations for further analysis and details [124,125]. In those that do not smoke many factors may play a role in development of lung cancer, not the least of which is ETS although the risks are much lower than in smokers and have proven difficult to estimate [126,127].

Tobacco smoke contains between 2,000 and 4,000 agents [128] that may exert toxic effects at different levels within the respiratory system and indeed distribute themselves, depending on their physical characteristics, at varying levels down the bronchial tree [129,130]. These compounds consist of a wide range of both organics and inorganics. In addition, volatile organic compounds (VOC's) in particular, some of which are naturally occurring in the tobacco plant while others such as asbestos and glass fibers occur as additives during the manufacturing process [131] are present. Whether from direct inhalation of tobacco smoke or secondhand smoke (ETS), as a first line of defense, the pulmonary surfactant is exposed primarily to inhaled gas-phase materials from tobacco or marijuana smoke and, to a lesser degree depending on the route, the particulates within the smoke as they are distributed within the airways. Thus, the cellular reaction to smoke exposure will depend to a great degree on the tissue level, the exposure material, either particulate or gas phase, and the effects of a complex mix of toxins on cellular metabolism. Since this review is focused on the pulmonary surfactant, we will assume here that components of the smoking process reach the terminal respiratory units and indeed this would seem to be the case, particularly for the gas phase constituents although particulate deposition in the airways, including the alveolar ducts and the entrance to alveoli, probably occurs [132]. The entire deposition process is very complex with the airways accumulating very specific sizes and types of particles [133,134]. Some of the smallest particles may even penetrate the mucosal epithelium, entering the pulmonary interstitium [135]. Thus, the effect on the pulmonary surfactant and indeed the entire lung may vary widely depending on the level and the type of contaminant. In this regard the actual physical characteristics of the surfactant, disregarding the contaminants' effects on synthesis and/or secretion, may be altered by many factors including subphase calcium [136], free oxygen radicals [137] and plasma proteins [138]. Consequently, the components of smoke may be expected to alter the surface tension lowering capabilities of pulmonary surfactant.

Studies of the direct effects of tobacco smoke on pulmonary surfactant have been few. Several older studies [139,140] suggest smoke may interact directly with surfactant and the ability to generate low surface tension may be impaired [140,141]. Higenbottam (1989) used a Wilhelmy balance to investigate if surface tension was altered in the presence of complete or filtered smoke [142]. While the smoke gas phase did not appear to change the hysteresis of the surface tension-area curve, whole smoke was surface-active, suggesting the particulates in smoke may have a detergent-like action. The majority of other studies have compared the characteristics of lung function or lung lavage in smokers and non-smokers. Tobacco smoke exerts a wide spectrum of biological effects on the lung and cells of the lung airways, including DNA damage [143], DNA single strand breaks in cultured human lung cells [144], bronchoconstriction associated with increased thromboxane levels [145], development of emphysema [146] and COPD [147]. Smoke may also stimulate the proliferation of lung cells through a ligand-epidermal growth factor receptor mechanism activated by tumor necrosis factor converting enzyme and oxygen radicals [148]. It is therefore not surprising that, at the level of the distal airways and alveoli, cigarette smoke alters the milieu of the air exchange tissues and distal airways.

Smoking appears to reduce the overall recovery by endobronchial lavage of surface active material and phosphatidylcholine in particular [149], although more recent reports suggest this change may not be as great as originally thought [150-152]. A somewhat contradictory finding suggests an initial rise in alveolar surfactant levels may occur [153,154] which the authors attribute to the short-term moderate-smoker characteristics of the population sampled. While several studies have been unable to detect alterations in the general phospholipid profile in the lavage fluid, the complexity of the lavage material dictates that such findings be taken in a critical light. Zetterberg and colleagues (1995) detected a higher fraction of palmitoylmyristoyl phosphatidylcholine in smokers [155] but did not find any changes in other disaturated phospholipids, which may suggest that the surface activity in the lung is within normal ranges since it is predominantly disaturated phosphatidylcholine on which this characteristic relies. On the other hand, Subramaniam and colleagues (1995), in an experimental model of chronic cigarette smoke exposure in rats, did detect a reduction in bronchoalveolar lavage DSPC levels but not tissue DSPC, suggesting smoke exposure alters surfactant secretion from type II alveolar cells [156]. In one of the few studies of surface tension characteristics, these authors also observed a major increase in surface film compressibility, probably related to the reduced DSPC. Taken together, these results suggest that at least chronic smoke exposure reduces pulmonary surfactant phospholipid levels.

Some changes have also been documented in other phospholipids. Specifically increases in phosphatidylethanolamine and sphingomyelin levels have been noted [153,154] possibly indicating cellular damage as these phospholipids are more associated with cell membranes than the pulmonary surfactant. Hughes and Haslam (1990) also observed significant reductions in the levels of lung lining fluid cholesterol and the cholesterol:phospholipid ratio in smokers which may in part explain surface tension changes observed in the chronic smoke exposure model [156] as this neutral lipid (cholesterol) may have an important role in fluidity [157]. The possibility also exists that smoking alters pulmonary repair processes as smoke extract appears to inhibit TGF-β and fibronectin release at the level of the bronchial epithelium and may conceivably have similar effects within the distal air spaces [158]. Obviously many factors are in play in interpreting smoking effects on surfactant phospholipids and it is too simplistic to expect a single effect or causative factor. Furthermore, it is important to bear in mind the complex nature of the surfactant components and their interactions. In this regard it is important that evidence is beginning to appear that smoking may alter the content, activity or properties of the surfactant proteins important to the cycling and generation of surface tension [159]. Effects on the proteins will be discussed later.

In addition to elevated numbers of alveolar macrophages [160], smokers show a severalfold increase in alveolar cells in lavage fluid and an increase in the numbers of cells expressing MHC molecules, but an overall underexpression of MHC and cell adhesion factor (LFA-1) molecules in these cells of the respiratory tract [154]. These authors also noted an overloading of alveolar macrophages with black inclusions which had been observed previously [161,162] and may be related to reduced surfactant levels [149] as macrophages phagocytose spent surfactant [163]. In this regard it is interesting to note that surfactant has recently been shown to improve mucociliary clearance in a model of acute induced bronchitis [164]. These observations would appear to support the idea that a thin surfactant layer intervenes along the bronchial epithelium between the mucus gel layer and the periciliary fluid surrounding the bronchial epithelial cilia [165-167]. In fact in a model of acute bronchitis induced by SO_2 _gas exposure, surfactant may improve mucociliary clearance from the bronchial tree [164]. However in smokers, this mucus-surfactant layer appears to be cleared much less rapidly compared to non-smokers [168]. This may be due to reduced ciliary activity and clearance rates in smokers [169,170]. Alternatively, or probably in addition to this mechanism, tobacco smoke may induce overproduction of mucin through activation of an epidermal growth factor receptor mechanism [171]. In any case, whether due to hypersecretion of mucus or smoking-induced reduction of clearance rates particularly in the larger airways [172], the ultimate effect is to increase mucus levels, possibly through the transcription factor NF-κB [173], thereby altering the pulmonary airway environment and increasing susceptibility to diseases such as asthma, cystic fibrosis or bronchitis. While this mechanism may explain elevated mucus levels, the increased load induces an elevation in ciliary beat frequency until uncoupling occurs [174]. What overall effect this has on the intervening surfactant layer at this level of the bronchial tree is not clear as smoke-induced effects have not been examined and the interactions of surfactant and mucus await further study.

At the molecular level, little is known about how smoke components interact with the surfactant either in the alveolus or at higher levels in the bronchial tree. Subramaniam and colleagues (1996) observed elevated levels of albumin in rat bronchoalveolar lavage following chronic cigarette smoke exposure [175] which has implications for the ability of surfactant to generate low surface tension as blood proteins such as albumin and fibrinogen are known to be detrimental to generation of low tension [176]. In addition cigarette smoke contains many compounds that may generate free radicals. Generally two populations of free radicals are present. The first is located in the tar or particulate phase, is relatively stable and reacts with DNA [177]. In contrast, the free radicals within the gas phase are less stable and much more reactive than those of the tar phase. While these compounds undoubtedly are implicated in such diseases as lung cancer and emphysema [177], some evidence has appeared that they induce injury to the surfactant-producing type II cells in the alveolus. Work by Lannan and colleagues (1994) suggests that the oxidant-antioxidant balance in the airspaces is important for alveolar cell function as both whole and vapour phase cigarette smoke were observed to decrease cell attachment and proliferation [178] while reactive oxygen species (ROS) were elevated [179]. Cigarette smoke extract also potentially induces necrosis and apoptosis, in a concentration-dependent fashion in A549 cells [180] and bronchiolar epithelial cells [181]. However it should be noted that A549 lung tumor cells display distinct differences from typical lung type II alveolar cells [182].

In a limited series of experiments, freshly isolated rat alveolar type II cells appeared to undergo cell lysis after exposure to tobacco smoke [178]. The importance of the surfactant in maintaining a biologically advantageous environment has clear implications for surface tension levels in the alveolus. Ozone exposure reduces the ability of the surfactant to maintain tube patency in a capillary surfactometer [183], reduces lung compliance and alters phospholipid levels [184]. Further, recent evidence of oxidative damage specifically to bronchiolar epithelial cells and type II alveolar cells suggests smoke exposure induces DNA damage and lipid peroxidation [185]. This may have implications for surfactant protein function as evidence has recently appeared that SP-A and SP-D contribute significantly to protection of the lung from oxidative stresses associated with exposure to air pollutants, oxygen or other agents [186].

In addition to the effects described above, some preliminary studies suggest cigarette smoke or smoke by-products affect the proteins of the pulmonary surfactant. Bronchoalveolar lavage samples from smokers and non-smokers showed reduced levels of SP-A and SP-D, standardized to lavage phospholipid levels [159] by ELISA and using human monoclonal antibodies to each protein. In an animal model, smoke exposure dramatically reduced the levels of both SP-A and SP-B in bronchoalveolar lavage but neither tissue protein nor lung RNA levels were altered [175]. On the other hand serum levels of SP-A appear to be increased in smokers [187,188]. It is not clear what implication these observations have for the ability of alveolar surfactant to generate low surface tension but may reflect changes in the complex cycling of surfactant in the hypophase as well as changes in synthetic and/or secretion rates.

Many other constituents of tobacco smoke may potentially alter or harm the pulmonary surfactant or type II cells. Of particular note, nitrogen dioxide, another constituent of tobacco smoke and a major environmental urban pollutant [189], has been examined for its ability to alter pulmonary surfactant synthesis or secretion. The effects of this agent on these processes will be discussed in the following section.

## Smoke Effects on Synthesis and Secretion of Lung Surfactant

It is generally accepted that surfactant deficiency, due to the immature nature of the surfactant-producing type II alveolar cells, is the main causative factor in the development of respiratory distress in prematurely delivered infants [190]. However, evidence is beginning to appear that components of the pulmonary surfactant, including the proteins, may be altered in various other disease states including such diseases as cystic fibrosis [191], asthma [192-194], allergic alveolitis [195] and chronic obstructive pulmonary disease (COPD) [196]. The associated changes may be primary or secondary and probably reflect alteration of the synthesis, secretion or reuptake of surfactant. Indeed the well established link between COPD and smoking suggests that surfactant may be a target for tobacco-induced pulmonary diseases [197]. Considering that smoke, its various components and possibly particulate matter reach the alveolar ducts and alveoli themselves, it might be expected that it would affect the intracellular synthetic rate, release, and probably the reuptake of the surfactant. Surprisingly little information is available concerning the effects of smoke on these processes.

As noted above there is good evidence that smoking is associated with reduced surfactant phospholipids in the bronchoalveolar lavage [149]. This may reflect the ability of smoke to reduce the levels of DSPC secretion, as observed in a rat model [156]. Similarly using an in vitro system of adult rat type II alveolar cells, Wirtz and Schmidt (1996) observed that cigarette smoke inhibited the stimulated but not the basal release of radiolabelled phosphatidylcholine [198]. This may involve the intracellular assembly and delivery of the surfactant to the cell membrane as smoke exposure alters the levels, although it causes an increase, of a phospholipid binding protein, annexin I in both type II alveolar cells and lavage fluid [199]. Such an effect may be explainable as a compensatory mechanism since no measures of annexin binding capacity were done to define the activity of the protein after smoke exposure. On the other hand, smoke generated by burning polyurethane foam did alter tissue DSPC levels and reduced enzymatic activity of phospholipase A_2_, suggesting the pulmonary effects may vary according to the type and composition of the inhaled smoke [115]. Furthermore individual smoke constitutents such as NO_2 _may have different effects as this compound appears to increase both the incorporation of precursors into surfactant phospholipids through enzyme activation, particularly CTP:cholinephosphate cytidylyltransferase, which regulates phosphatidylcholine synthesis [200,201] and release of phosphatidylcholine in isolated rabbit type II cells [200,202]. In fact cellular damage may be connected to the release as LDH levels were correspondingly increased. Thus some evidence exists that smoke products have the ability to markedly alter intracellular synthetic and secretory rates, probably through effects on enzymatic conversion or transport of surfactant components within type II cells. However it is difficult to generalize as to the potential effects of any particular compound on surfactant synthesis and secretion.

## Smoke Effects on Lung Development

Despite early observations that smoke exposure during pregnancy actually hastens pulmonary maturation [203,204] which was ascribed to "adverse pregnancy conditions" [203], many recent studies have rather conclusively shown that smoking during pregnancy comes at a high cost. Prenatal smoke exposure has been shown to be associated with neonatal mortality [205], low birth weight/growth retardation [206] and an increase in the rate of spontaneous abortions [207]. In relation to the pulmonary system, impairment of infant breathing [208-211] and breathing control [212], retardation of lung growth [213], reduced flow rates in infants of smoking mothers [214,215] particularly in children who develop asthma [215] as well as increased airway responsiveness [216,217], a hallmark of asthma have been reported. Furthermore compression of the fetal lungs, reduction of fetal breathing movements, hypoxia [218,219] or placental insufficiency are among many factors that can be directly responsible for respiratory compromise in later life [211]. Maternal smoking and fetal exposure during pregnancy may be significant components of many of these causative factors which may persist, even without early postnatal smoke exposure [220,221] and induce pulmonary changes. Pulmonary cell apoptosis, hyperplastic bronchial smooth muscle and mesenchymal disorganization were reported in rat pup lungs exposed *in utero *to smoke [222]. In fact, some evidence suggests exposure to tobacco smoke components during pregnancy may play a much greater role in altered lung function than postnatal or childhood exposure [223]. These pulmonary changes appear to occur early in pregnancy as respiratory function in premature infants of smoking mothers is significantly reduced compared to premature infants of nonsmokers [224], indicating insult to the developing respiratory tissues does not occur only during the late stages of pregnancy. Such a stress-induced mechanism may indeed explain some of the observations as a glucocorticoid effect on pulmonary maturation may be instrumental in induction and regulation of lung development and several components of the pulmonary surfactant [55]. The ultimate effect of smoke exposure *in utero *and the postnatal period on the pulmonary surfactant is, however, not clear. In rat pups from smoke-exposed mothers and following postnatal sidestream exposure changes in dynamic compliance, reactivity to methacholine [225], and biochemical changes in total lavage phospholipids and decreased lavage SP-A levels were detected [226]. However in the latter study, these changes were not reflected in corresponding changes of the biophysical properties of the lavage surfactant as measured on a pulsating bubble surfactometer.

While many agents in smoke may be detrimental to fetal development, some evidence supports a direct effect of nicotine on lung development suggesting that nicotine is capable of altering lung maturation. Nicotine crosses the human placenta with only minimal biotransformation to cotinine [227]. It accumulates in fetal blood, maternal milk and amniotic fluid [228] despite increased nicotine clearance during pregnancy [229] and may result in the fetus being exposed to even higher levels than those of the smoking mother [230]. This material appears to accumulate in several fetal tissues including the respiratory tract and urinary bladder [231]. However, what exactly the nicotine does at the level of the lung is not clear. Maritz and colleagues, in several morphological studies appear to have demonstrated that nicotine exposure during pregnancy decreased the ratio of type I:type II cells in the neonatal lung as well as increasing the lamellar body content of the latter cell type [232]. As the type II cells are the precursors to the type I cells in injured lung [233] and both probably share a common progenitor cell type in the fetal lung [234], such a shift suggests a major effect on differentiation of the alveolar epithelium. In addition the accumulation of lamellar bodies further suggests that secretion of surfactant may be inhibited following nicotine exposure although this may be related to an overall alteration in glucose metabolism observed in fetuses exposed to this drug *in utero *[235]. Changes have also been described in connective tissue components of lung and lung airways associated with nicotine treatment during pregnancy. At the alveolar level, Maritz and Woolward (1992) observed a reduction in elastic tissue content and suggested this may potentially make infants exposed to nicotine more susceptible to emphysema [236]. Quantification of nicotine effects on developing fetal rat lung [237] suggests an overall retardation of growth and maturation, and an increase in a destructive index previously associated with lung emphysema of adult smokers [238]. Nicotine also induces an up-regulation of α7-nicotinic acetylcholine receptors (nAChR's) in lung, an increase in collagen and elastin deposition in the airways as well as an increase in alveolar type II cell numbers in fetal monkey lung [239,240]. The authors interpret these findings, in light of the degree of lung hypoplasia detected, to mean that nicotine exposure *in utero *induces altered lung mechanics in the fetus. However as they note, multiple binding sites for nicotine in developing lung, including the neuroendocrine cells, probably signifies a complex, multidimensional effect on the lung including such complex mechanisms as regulation of dichotomous branching, connection tissue interactions and muscle induction [239]. Indeed the neuroendocrine cells which phenotypically resemble small cell lung carcinomas appear also to be binding sites for the tobacco-specific carcinogen NNK through a MAP kinase/c-myc pathway via nAChR's and may provide several alternative routes whereby lung development may be altered through a paracrine serotonin pathway [241,242].

While these studies overwhelming support the detrimental role for tobacco smoke exposure on prenatal and postnatal lung development and the overall well-being of the fetus, paradoxical evidence alluded to at the first of this section suggests that smoke-exposed fetuses display a state of advanced lung maturity [203,204]. Such an effect is supported by evidential measures of amniotic fluid Lecithin/Sphingomyelin (L/S) ratio and saturated phosphatidylcholine levels [243] which would suggest an acceleration of maturation by approximately one week. These same investigators observed an elevation of amniotic fluid cortisol, providing strong support for the contention that the smoke-induced effect is secondary to fetal stress and hypoxia [244,245], and that glucocorticoids play an important role in stimulation of lung maturation [103]. Nevertheless recent evidence suggests a direct nicotine effect as *in vitro *exposure of embryonic lung explants induced morphogenetic branching and enhanced expression of surfactant proteins A and C and the Clara cell-specific marker CC10 [246]. This action may be related to the presence of the nACHr's in lung and it is relevant to note again that these receptors appear to be present in airway epithelial cells as well as the pulmonary neuroendocrine cells and type II cells [239]. Nevertheless it has not been determined what role activation of these receptors may play in the latter cell type and if it relates in any way to the regulation of surfactant synthesis and secretion.

## Conclusion

Tobacco smoking, primarily through the use of cigarettes, was at one time a pervasive and accepted social habit. While use has declined to some extent in selected areas and the "acceptedness" of smoking, particularly in indoor environments, has similarly declined, exposure to tobacco smoke still remains a major health risk particularly in developing countries [247]. Inhalation of tobacco smoke has major effects on the lung, one being the induction of lung cancer. Subtler effects on the lung and the pulmonary surfactant are probable as the extant research literature supports the detrimental nature of smoke on many lung processes. The pulmonary surfactant in particular which forms the first line of defense against inhaled materials may be adversely affected but evidence of such an effect has not been examined in great detail. Additional research in this area is clearly required. Part of the reason for this lack is likely the complex nature of the smoke generated by cigarettes which as a result makes it difficult to select likely candidates in this complex cocktail of volatile and particulate matter for study. Nonetheless, many of the major components such as nicotine and nitrogen dioxide have documented effects, although many details still remain to be elucidated.

With regard to the developing fetus and fetal lung where the critical nature of the developmental process is underscored by the mortality levels due to respiratory distress, little question remains that smoking during pregnancy is detrimental. Altered fetal intracellular enzyme levels, receptor binding proteins and surfactant proteins are among some of the initial findings. Furthermore these changes are reflected in altered lung mechanics in the newborn which may extend into later periods of development and may compromise normal expansion and compliance during childhood. Clearly more research and application of markedly more sophisticated techniques as well as development of *in vitro *models will be necessary to establish smoke effects on lung function. This will enable characterization of the mechanisms by which agents in smoke potentially alter lung maturation, the developing respiratory tissues, and particularly the pulmonary surfactant.

**Figure 3:**
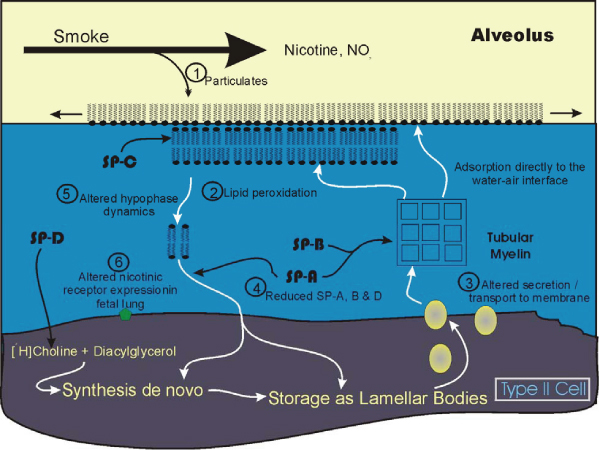
**Examples of some potential sites where smoke exposure may alter surfactant metabolism**. 1: Particulates may have a direct detergent-like effect on the surface monolayer. 2: Lipid peroxidation induced by smoke exposure. 3: Transport of surfactant-specific phospholipid and altered secretion of lamellar bodies. 4 and 5: Reduced levels of SP-A and SP-D may affect hypophase processing directly or indirectly or alter SP-D regulation of DPPC synthesis. 6: Nicotine may alter receptor expression in fetal lung.

## Competing interests

The authors declare that they have no competing interests.
